# Inability to get up after falling, subsequent time on floor, and summoning help: prospective cohort study in people over 90

**DOI:** 10.1136/bmj.a2227

**Published:** 2008-11-17

**Authors:** Jane Fleming, Carol Brayne

**Affiliations:** 1 Department of Public Health and Primary Care, University of Cambridge, Institute of Public Health, Cambridge CB2 0SR

## Abstract

**Objectives** To describe the incidence and extent of lying on the floor for a long time after being unable to get up from a fall among people aged over 90; to explore their use of call alarm systems in these circumstances.

**Design** 1 year follow-up of participants in a prospective cohort study of ageing, using fall calendars, phone calls, and visits.

**Setting** Participants’ usual place of residence (own homes or care homes), mostly in Cambridge.

**Participants** 90 women and 20 men aged over 90 (n=110), surviving participants of the Cambridge City over-75s Cohort, a population based sample.

**Main outcome measures** Inability to get up without help, lying on floor for a long time after falling, associated factors; availability and use of call alarm systems; participants’ views on using call alarms to summon help if needed after falling.

**Results** In one year’s intensive follow-up, 54% (144/265) of fall reports described the participant as being found on the floor and 82% (217/265) of falls occurred when the person was alone. Of the 60% who fell, 80% (53/66) were unable to get up after at least one fall and 30% (20/66) had lain on the floor for an hour or more. Difficulty in getting up was consistently associated with age, reported mobility, and severe cognitive impairment. Cognition was the only characteristic that predicted lying on the floor for a long time. Lying on the floor for a long time was strongly associated with serious injuries, admission to hospital, and subsequent moves into long term care. Call alarms were widely available but were not used in most cases of falls that led to lying on the floor for a long time. Comments from older people and carers showed the complexity of issues around the use of call alarms, including perceptions of irrelevance, concerns about independence, and practical difficulties.

**Conclusions** Lying on the floor for a long time after falling is more common among the “oldest old” than previously thought and is associated with serious consequences. Factors indicating higher risk and comments from participants suggest practical implications. People need training in strategies to get up from the floor. Work is needed on access and activation issues for design of call alarms and information for their effective use. Care providers need better understanding of the perceptions of older people to provide acceptable support services.

## Introduction

The risk of falling increases with age and declining muscle function reduces the ability to get up. If an older person is unable to get up off the floor after a fall, the attendant risks of any fall are far greater because of the complications that can ensue from lying on the floor for a long time—for example, pressure sores (often exacerbated by unavoidable incontinence), carpet burns, dehydration, hypothermia, pneumonia, and even death.^1^ Attending older people who have fallen is a major component of emergency service duties for ambulance crews,^2^ and, even for the high proportion of these people not transferred to hospital, this inability to get up has a poor prognosis in terms of the risk of injury in a subsequent fall,^3^ admission to hospital,^1^ and mortality.^1^ ^4^ Lying on the floor for a long time is thus one of the most serious consequences of a fall, and, with population growth already fastest among the oldest old (people aged ≥90), there is a need for population based estimates of the extent of this problem in advanced old age.

Assistive technology might have a role in the care of vulnerable people who are often alone,^5^ ^6^ ^7^ ^8^ and devices such as call alarm systems are increasingly available to older people in the United Kingdom. A few studies in the United States have found that such personal emergency response systems might be cost effective in reducing hospital admissions,^9^ ^10^ but uptake and adherence are low.^11^ ^12^ In our prospective study of falls among the oldest old we sought to quantify lying on the floor a long time and explored the extent to which such alarms were used.

## Methods

Data were collected on the immediate consequences of falls among participants of a population based study—the Cambridge City over-75s Cohort (CC75C). The methods have been described in detail elsewhere for the cohort overall (www.cc75c.group.cam.ac.uk)^13^ and for its survey at year 17, when all participants were aged over 90, which focused on falls.^14^ ^15^

The longitudinal cohort study of older old people initially recruited participants through general practices in the 1980s, when they were all aged 75 or over. Repeated surveys since baseline have gathered data on a range of variables including sociodemographics, physical and mental health, function, and detailed cognitive assessment^16^ that included the mini-mental state examination.^17^ All those who took part in the 2002-3 survey (90 women and 20 men) were followed up in a prospective study of falls for one year or until death if sooner. We gathered details of each fall either when falls were reported by telephone or, after we received a weekly report on falls by post, when the project nurse made a follow-up visit or phone call. Whenever possible, information was sought both from the study participant and from any proxy informant. Data recorded after each fall included whether the individual who fell had been able to get up without help, how long they were on the floor, any injuries, and whether they called for assistance. We used the classifications of Tinetti et al to ask about needing help to rise,^1^ and our study protocol followed the wording and categorisation of time on the floor from Nevitt et al.^18^ Measurement of injuries and hospital admissions will be published elsewhere; briefly, injuries were categorised as major or minor after the widely used definitions applied in the major Scandinavian surveillance studies.^19^ ^20^ We classed injuries that resulted in pain for over two weeks as “serious minor injuries,” according to the categorisation of Lord et al,^21^ and grouped them with major injuries as “serious injuries.” Follow-up procedures kept track of dates and reasons for hospital admissions and discharges and for moves into long term care. We used the categorisation “severe cognitive impairment” for participants with scores 0-17 on the mini-mental state examination or with diagnosed dementia. Terms for place of residence reflect the UK context: supported settings include “sheltered” housing schemes with live-in or visiting wardens on call as well as long term care institutions, ranging from homes providing personal care to nursing homes. We classified people “living in a house, flat, or granny flat” as “community dwelling.”

### Analysis

We performed analyses in Stata 9 and explored cross tabulations of descriptive data. We compared those who did or did not report falls during follow-up by examining differences in values of the continuous variable age using Student’s *t* test and differences in proportions of binary and ordered categorical variables using Pearson χ^2^ test, with Fisher’s exact test where appropriate, and the score test for trend. Bivariate and multiple variable associations with not being able to get up unaided after falling and with lying on the floor for over an hour were quantified with logistic regression and with Cox regression for one time dependent variable. We adjusted crude odds ratios and hazard ratios for age, sex, and mobility using the only factor common to the different models selected by forwards and backwards stepwise regressions—climbing less than one flight of stairs a day. We recorded subjective comments of participants and relatives and coded the qualitative data from these verbatim transcripts using framework analysis methods to identify emergent themes concerning the use of call alarms and summoning help.

## Results

### Characteristics of the study sample

Of the surviving participants from the original Cambridge study, 110 (84%) took part in the falls survey and follow-up (age 91-105, median 94). Sixty six (60%) were reported to have fallen at least once during the year after interview.^14^ ^15^ Table 1 describes characteristics of these 56 women and 10 men alongside the full sample and those with no reported falls during follow-up. Participants were predominantly frail—most were unable to climb stairs, nearly a third were unable to walk outdoors, one in 10 were housebound, and about one in three were severely cognitively impaired. Fewer than half, however, were living in institutional or sheltered settings and more than a fifth were still able to walk around their local neighbourhood. Two thirds of the people living in the community lived alone, and all those in sheltered housing were in single flats. More of those who reported falling during the follow-up year tended to have worse cognitive and mobility impairment than the others, but their differences reached significance only in the history given of recalled falls in the preceding year.

**Table 1 tbl1:** Characteristics of study sample. Figures are numbers (percentages) of participants unless stated otherwise

	All participants (n=110)	Participants with ≥1 fall reported (n=66)	Participants with no fall reported (n=44)	P value*
Age (years):				
Mean (SD)	94.4 (2.4)	94.6 (2.6)	94.2 (1.9)	0.4
Median	93.8	93.8	93.7	—
Interquartile range	92.7-95.8	92.7-96.0	92.7-95.6	—
Range	91.6-105.8	91.8-105.8	91.6-99.3	—
Women	90 (82)	56 (85)	34 (77)	0.3
Left school aged ≥15 years	43 (39)	28 (42)	15 (34)	0.4
Non-manual social class†	51 (48)	30 (47)	21 (50)	0.8
Place of residence:				
Community	62 (56)	35 (53)	27 (61)	0.4
Sheltered housing	19 (17)	13 (20)	6 (14)	
Care home/long stay ward	29 (26)	18 (27)	11 (25)	
Living alone:				
Community	43/62 (69)	26/35 (74)	17/27 (63)	0.3
Sheltered housing	19/19 (100)	13/13 (100)	6/6 (100)	0.5
Maximum walking distance:				
Unable to walk around local area	84 (76)	52 (79)	32 (73)	0.5
Unable to walk outdoors	34 (31)	19 (29)	15 (34)	0.6
Use of walking aid:				
Not needed outdoors	13 (12)	5 (8)	8 (18)	0.6
Needed aid outdoors	86 (78)	54 (82)	32 (73)	
Never goes outdoors	11 (10)	7 (11)	4 (9)	
Not needed indoors	38 (35)	22 (33)	16 (36)	0.4
Needed aid indoors	71 (64)	44 (67)	27 (61)	
Bedridden	1 (1)	0 (0)	1 (2)	
Cannot climb stairs/<1 flight/day	72 (65)	47 (71)	25 (57)	0.1
Severe cognitive impairment‡	35 (32)	24 (36)	11 (25)	0.2
Falls in previous year§:				
None recalled	46 (42)	23 (35)	23 (52)	<0.001
1 recalled fall	26 (24)	9 (15)	17 (39)	
≥2 recalled falls	37 (34)	33 (50)	4 (9)	

*For difference between participants with ≥1 and no reported fall(s).

†Data missing for four, two each with and without reported falls.

‡Min-mental state examination score 0-17 or diagnosed dementia

§Data missing for one.

### Falling in different residential settings

During follow-up at least one fall was reported for 56% (35/62) of the participants living in the community, 68% (13/19) of those living in sheltered housing, and 62% (18/29) of those in institutional settings (residential care, nursing home, or hospital) at the time of interview. There were 265 reported falls in total as most people fell at least twice.^15^ This was not always in the same setting as at interview as some participants who were originally in their own homes were subsequently in hospital or care homes when they fell. Falls in the community accounted for 45% (120/265) of all falls reported; the remaining 23% (62/265) and 31% (83/265) of falls happened in sheltered housing schemes and institutional settings respectively.

### Falling alone

In one year’s intensive follow-up, over half (144/265, 54%) of fall reports described the participant as being found on the floor. Table 2 shows the proportions of falls that happened when the person who fell was on their own: 82% (217/265) overall but as high as 94% (58/62) in sheltered accommodation. In terms of people who fell, rather than falls, 88% (58/66) were alone when at least one of these falls happened.

**Table 2 tbl2:** Falls that occurred when participant was alone, inability to get up, and time on floor by place of residence. Figures are numbers (percentages) of falls by setting

	Community (n=120)	Sheltered housing (n=62)	Institutional settings (n=83)	All (n=265)
Alone when fell	93 (78)	58 (94)	66 (80)	217 (82)
Needed help to get up	52 (43)	41 (66)	83 (100)	176 (66)
Time on floor (hours)				
<1	97 (81)	40 (65)	72 (87)	209 (79)
≥1, excluding unknown	16 (13)	17 (27)	7 (8)	40 (15)
Unknown	7 (6)	5 (8)	4 (5)	16 (6)

### Inability to get up from the floor

Not being able to get up was equally common whether a fall was witnessed or occurred alone. Everyone who fell in an institutional setting, (66%, 41/62) who fell in sheltered housing, and (43%, 52/120) who fell in community settings needed help to get up after a fall (table 2).[Table tbl2] Again, considering not only the proportions of falls when this was a problem but also the proportion of people who fell who were affected, the latter was higher: in two thirds of all the falls reported the person who fell was unable to get up without help (176/265, 66%), but 80% (53/66) of the participants who fell had difficulty getting up from at least one fall.

### Lying on the floor for a long time

Fifteen per cent (n=40) of all reported falls in different settings resulted in the person lying on the floor for an hour or more (table 2).[Table tbl2] The length of time on the floor was unknown for a further 6% (n=16). When we consider people who fell, rather than falls, these proportions are even higher: 30% (n=20) of those who fell were on the floor for an hour or more on at least one occasion, and for a further 9% (n=6) the maximum time was unknown (data not tabulated).

The length of time on the floor after falling depended both on ability to get up after falling and on whether there was any help at hand. Table 3 details reported time on the floor for all falls, falls with no one present, falls when help was needed to get up, and falls when such help was needed but no one was there at the time of the fall.[Table tbl3] Increasing proportions of falls led to longer times on the floor across each of these categories. While 43% (114/265) of all falls led to five minutes or less on the floor, those who needed help to get up were helped up in less than five minutes in just a quarter of these falls (46/176, 26%), and for those who were alone when they fell and could not get off the floor, assistance to help them up arrived in less than five minutes for only 17% (25/143) of such falls. Even if the person who fell was not alone when the fall happened, those who could not get up unaided could be on the floor for a considerable time because of the difficulty those present had in helping them up: in 10 such cases this took between five minutes and an hour.

**Table 3 tbl3:** Time on floor after fall during one year follow-up. Figures are numbers (percentages) of falls

	All falls (n=265)	Participant alone (n=217)	Participant unable to get up without help (n=176)	Participant alone and unable to get up without help (n=143)
<5 minutes	114 (43)	78 (36)	46 (26)	25 (17)
5 minutes-<1 hour	95 (36)	85 (39)	78 (44)	68 (48)
1-2 hours	14 (5)	14 (6)	14 (8)	14 (10)
>2 hours	26 (10)	26 (12)	26 (15)	26 (18)
Unknown	16 (6)	14 (7)	12 (7)	10 (7)

All the incidents in which people lay on the floor for over an hour arose from unwitnessed falls, and 28% (40/143) of unwitnessed falls led to the person lying on the floor for an hour or more. This proportion rose to over a third when we included falls with unknown floor times. This overall 35% (50/143), which includes relatively few cases of a long time lying on the floor in care homes, is far lower than the high prevalence in such situations among residents in sheltered housing (44-49% excluding or including lying on floor for unknown time) and participants living in the community (42-56%) (fig 1).

**Figure fig1:**
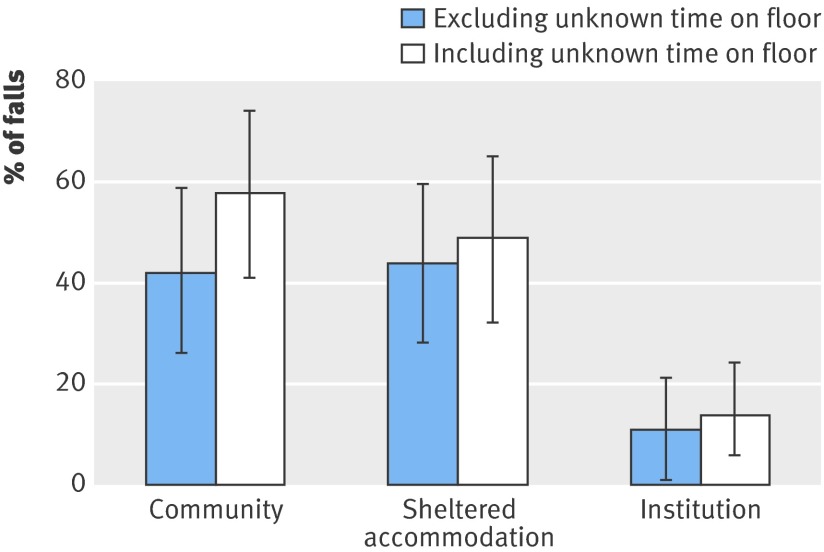
**Fig 1** Lying on the floor for over an hour after falling (143 falls in people aged >90 who were alone and unable to get up). Proportions with 95% confidence intervals

### Factors associated with inability to get up and lying on the floor for a long time 

Table 4 describes the participants who were unable to get up after a fall (n=53), those who were on the floor for at least an hour (n=20) on at least one occasion during the follow-up year, and the prevalence of injury, admission to hospital, and admission to long term care.[Table tbl4] Injuries can be both a cause and a result of lying on the floor for a long time, which can be viewed not only as a possible outcome of falling but also as a predictor of further sequelae such as admissions to hospital and care home. Table 4 shows the associations found between all these factors—descriptive characteristics and potential longer term sequelae—and the two possible results of falling detailed above—being unable to get up again without help and consequently remaining on the floor for over an hour.

**Table 4 tbl4:** Factors associated with inability to get up and lying on the floor for over an hour after falling, with unadjusted and adjusted odds ratios (95% confidence intervals)

	Unable to get up without help after ≥1 fall				Lying on floor for at least 1 hour after ≥1 fall		
	No of participants (n=53)	Unadjusted (95% CI)	Adjusted* (95% CI)		No of participants (n=20)	Unadjusted (95% CI)	Adjusted* (95% CI)
Mean (SD) age (years)	94.9 (2.8)				94.5 (1.9)		
For each additional year of age	–	1.4 (1.0 to 2.1)	1.7 (1.0 to 2.8)		–	1.0 (0.8 to 1.2)	1.0 (0.8 to 1.2)
Women *v* men	48 (91)	6.0 (1.4 to 25.5)	2.5 (0.4 to 15.6)		18 (90)	1.9 (0.4 to 9.8)	1.5 (0.3 to 8.7)
Left school aged ≥15 *v* ≤14	21 (40)	0.6 (0.2 to 1.9)	0.4 (0.1 to 2.0)		8 (20)	0.9 (0.3 to 2.5)	0.9 (0.3 to 2.6)
Non-manual *v* manual social class†	23 (45)	0.7 (0.2 to 2.4)	0.5 (0.1 to 2.3)		11 (55)	1.6 (0.6 to 4.7)	1.6 (0.6 to 4.8)
Place of residence:							
Supported setting (sheltered/institution) *v* community	30 (57)	15.7 (1.9 to 129.0)	7.1 (0.6 to 76.1)		10 (50)	1.2 (0.4 to 3.4)	0.9 (0.3 to 3.0)
Living alone *v* with spouse/other family (excluding institutions)	31/35 (89)	4.8 (1.1 to 22.3)	10.1 (1.0 to 99.0)		13/14 (93)	4.0 (0.5 to 35.5)	5.9 (0.5 to 77.6)
Maximum walking distance:							
Unable *v* unable to walk around local area	46 (87)	7.7 (2.0 to 29.6)	5.0 (1.0 to 26.7)		18 (90)	3.2 (0.6 to 15.8)	3.0 (0.6 to 15.9)
Unable *v* unable to walk outdoors	18 (34)	6.2 (0.7 to 51.3)	3.7 (0.2 to 68.6)		4 (20)	0.5 (0.2 to 1.8)	0.4 (0.1 to 1.5)
Use of walking aid:							
Needed *v* not needed outdoors (excluding unable to walk outdoors)	44/46 (96)	6.6 (1.0 to 44.9)	2.4 (0.2 to 23.6)		17/18 (94)	1.8 (0.2 to 17.7)	1.4 (0.1 to 15.5)
Needed *v* not needed indoors	39 (74)	4.5 (1.3 to 15.9)	1.7 (0.3 to 8.7)		16 (80)	2.6 (0.7 to 8.9)	2.6 (0.6 to 10.3)
Cannot climb stairs or <1 *v* ≥1 flight/day	44 (83)	16.3 (3.7 to 71.3)	16.6 (3.1 to 87.7)		16 (80)	1.9 (0.6 to 6.8)	1.7 (0.5 to 6.7)
Severe cognitive impairment‡ *v* moderate/no impairment	23 (43)	9.2 (1.1 to 76.0)	4.1 (0.4 to 46.3)		13 (65)	5.9 (1.9 to 18.5)	8.1 (2.1 to 31.0)
Recalled falls in past year§:							
≥1 *v* none	37 (71)	3.9 (1.1 to 14.0)	2.7 (0.5 to 13.9)		13 (65)	1.0 (0.3 to 3.1)	0.9 (0.3 to 2.9)
≥2 *v* none	31 (60)	8.3 (1.6 to 43.8)	5.3 (0.6 to 44.6)		11 (55)	1.1 (0.4 to 3.6)	1.0 (0.3 to 3.5)
Injuries sustained in falling:							
Any *v* none	40 (75)	4.9 (1.4 to 17.7)	3.0 (0.6 to 14.6)		17 (85)	3.6 (0.9 to 14.2)	3.5 (0.9 to 14.4)
Serious *v* non-serious/none	13 (25)	3.9 (0.5 to 32.9)	3.2 (0.3 to 42.7)		8 (40)	4.4 (1.3 to 15.4)	4.2 (1.2 to 14.8)
Serious *v* none (excluding non-serious)		8.0 (0.9 to 73.4)	6.6 (03. to 170.8)			8.0 (1.6 to 40.3)	7.4 (1.3 to 41.1)
Hospital admissions:							
All cause hospital admission *v* no admission	31 (59)	7.8 (1.6 to 38.5)	17.9 (2.3 to 140.3)		13 (65)	2.4 (0.8 to 7.2)	2.4 (0.8 to 7.3)
Fall related hospital admission *v* no fall related admission	24 (45)	9.9 (1.2 to 82.0)	21.1(1.9 to 230.5)		12 (60)	3.8 (1.3 to 11.5)	4.0 (1.3 to 12.3)
Moving to institutional care**:							
Moved into care within 1 year *v* not moved into care	11/38 (29)	—††	—††		5/14 (36)	2.9 (0.7 to 11.6)	2.4 (0.5 to 11.0)
Moved into care by study end censoring *v* not moved	15/38 (39)	—††	—††		8/15 (53)	3.4‡‡ (1.2 to 9.5)	2.3‡‡ (0.8 to 7.0)

*Model selected by multiple variable regression adjusted for age, sex, and mobility (climbing less than flight of stairs/day); hospital admissions adjusted only for mobility because of low numbers).

†Data missing for two.

‡Mini-mental state examination score 0-17 or diagnosis of dementia.

§Data missing for one.

**Excludes 15/66 who fell who were already in long term institutional care.

††No one who was able to get up unaided moved into care within 1 year follow-up nor by end of study censoring.

‡‡Hazard ratio.

Univariate analyses showed that many factors were associated with difficulty in rising from the floor, the important sociodemographic descriptors being age, sex, and residential status. Women were six times more likely than men to have difficulty getting up. Residents of any supported living setting—such as sheltered accommodation, residential care, or nursing homes—had a 16-fold increased odds of being unable to get off the floor without help; no one who fell in a care home was able to get up unaided (data not shown). Those who lived alone in the community or in sheltered housing were particularly unlikely to be able to get up after falling: the almost fivefold crude odds doubled to an adjusted odds ratio over 10. Reported mobility was consistently associated with inability to get up, limited walking distance, and the need for walking aids, conferring about fourfold to eightfold increased odds, and the odds ratio doubled again to 16-fold for climbing less than a flight of stairs a day or being unable to manage stairs at all. Regression modelling identified this mobility descriptor as the most consistent predictor for this serious consequence of falls, outweighing the effects of cognitive impairment and recalled falls in adjusted models. Exclusion from reported mobility analyses of those who cannot walk at all had no effect on the significance of risk estimates for either outcome.

Severe cognitive impairment was highly significantly associated with lying on the floor for a long time and, unlike the reduction of effect in relation to getting up unaided, adjustment for mobility, age, and sex strengthened this finding: adjusted odds ratio 8.1 (95% confidence interval 2.1 to 31.0). Living alone quadrupled the odds of lying on the floor for a long time but, with wide confidence intervals from the smaller sample in this analysis, even the higher adjusted estimate did not reach significance: 5.9 (0.5 to 77.6). No other descriptive characteristics predicted the length of time on the floor.

There were marked associations with other potential sequelae of falling for both being unable to get up and for lying on the floor for a long time. Any degree of injury was common, regardless of time on the floor, and increased the crude odds of needing help to get up fivefold, though this was not a significant factor after adjustment. Serious injury, however, was consistently associated with lying on the floor for a long time, regardless of adjustment. Of the 20 people who were reported to be on the floor over an hour after falling, 60% (12) had a fall related hospital admission during the follow-up year (4.0, 1.3 to 12.3). Twenty nine per cent of those who had been unable to get up from the floor (11/38) moved into long term care within a year of interview and 39% (15/38) by the end of study censoring. The equivalent figures for those who remained on the floor for over an hour were 36% (5/14) and 53% (8/15), giving a threefold increased odds of admission to a care home, though the risk estimate did not reach significance in this smaller sample (n=51 not living in care who fell) until the censoring, conducted when everyone had completed their year since interview (crude hazard ratio 3.4 (1.2 to 9.5), adjusted 2.3 (0.8 to 7.0)).

### Use of call alarm systems to summon help

Many of those in the study population had call alarm systems. About a third each had either a personal alarm linked to a call centre when activated, usually a pendant, or call bells installed in their room or flat (everyone in a care home and most in sheltered schemes), and 12% (13) had both. Table 5 shows the variation across different residential settings.[Table tbl5] Of those living alone in the community or sheltered accommodation, 70% (57/81) had some form of call alarm.

**Table 5 tbl5:** Access to call alarm systems in different residential settings. Figures are numbers (percentages) of participants

	Community dwelling (n=62)	Sheltered housing (n=19)	Institutional care (n=29)	All (n=110)
None	24 (39)	0	0	24 (22)
Personal call alarm system	36 (58)	2 (11)	0	38 (36)
Call bell in flat/room/by bed	1 (2)	5 (26)	29 (100)	35 (32)
Both systems	1 (2)	12 (63)	0	13 (12)

In 95% (209/219) of falls that occurred when the individual was alone, and in 99% (141/143) of falls in those who could not get up when they fell alone, the person had some form of call alarm system. In 80% (113/141) of these falls, however, the person who fell alone did not use their call alarm to summon help. This was most often the case in institutional settings (94%, 62/66) but was also so for most falls both in the community (78%, 28/36) and in sheltered accommodation (59%, 23/39). Of these 141 falls, 38 resulted in lying on the floor for over an hour, despite an installed alarm system, and in 97% of these “long lies” (37/38) the person who fell alone did not use their alarm to summon help.

Figure 2 shows the relation between summoning help and the length of time on the floor. Lying on the floor for an hour or more occurred in only one or perhaps two out of the 28 falls in which a call alarm was activated by someone unable to rise when they fell on their own. One arose from a resident in a care home taking over an hour to crawl round her bed to reach the call bell after falling as she got out of bed. In the other case, the only incident known to involve alcohol, the time on the floor could not be established but the mobile warden team who answered the call presumed it was a long time.

**Figure fig2:**
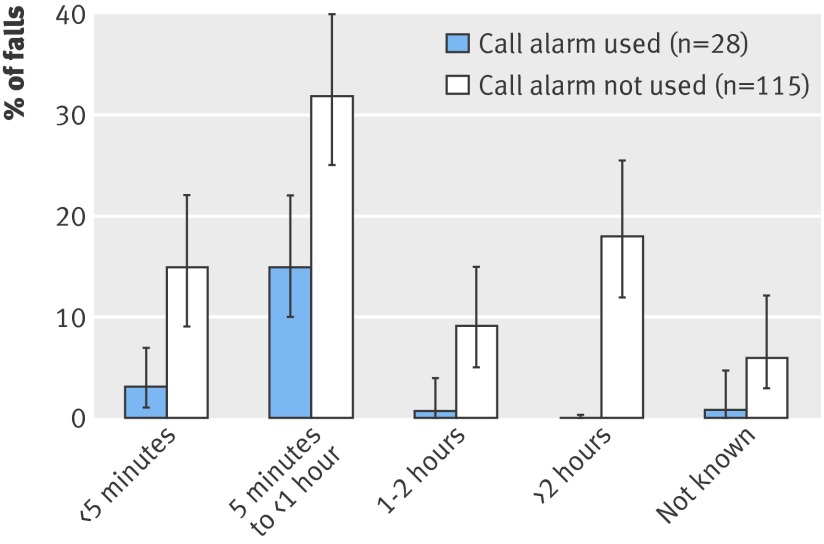
**Fig 2** Time on floor according to use of call alarm (143 falls in people aged >90 who were alone and unable to get up). Proportions with 95% confidence intervals

### Factors influencing use of call alarms

Several themes emerged from analysis of comments made by participants and carers about why call alarms were not used. The box provides examples illustrating the range of their attitudes and abbreviated scenarios describing how the person quoted coped when they did not call for help. Barriers to using alarms arose at several crucial stages: not seeing any advantage in having such a system, not developing the habit of wearing the pendant even if the system was installed, and, in the event of a fall, not activating the alarm—either as a conscious decision or as a failed attempt.

**Attitudes towards the use of call alarms to summon help**

**Not having a call alarm**

“My niece is only next door. I can bang on the wall if I need to call help.”

Daughter’s comments: “She refuses to have a call alarm because she thinks it would keep going off by mistake. She is worried enough about the string pull alarms in each room [sheltered housing scheme] and often won’t turn on the kitchen or bathroom lights in case she pulls the wrong cord by mistake.”

**Having one but not wearing it**

“I have got one but I don’t have to wear it yet, I just hang it on the back of the chair there.”

“I wasn’t wearing my pendant. I don’t usually wear it . . . It was quite a struggle to get up. It took about half an hour. My sister pushed me across to the sofabed and we used that to help get me up . . . I’ve been thinking after you asked last time, maybe I should wear it when my sister is out.”

“I’d already taken it off ready for bed and put it on the bedside table so then I couldn’t reach it.” [Lost balance getting undressed, was on the floor all night until next morning finally attracted the attention of someone delivering a newspaper by calling through the doorbell intercom]

**Wearing but choosing not to use it**

“I wanted to be able to get up by myself. It took me a long time to get up but I did it in the end. It makes me annoyed if I have to have help.” [Fell bending down to pick up a letter at the door]

“I didn’t want to use the call alarm, although I was wearing it, for fear of being taken into hospital.” [Trying to stand up from the toilet, fell on her back] “I grabbed the portable arms round the loo when I lost balance but they tilted. It took quarter of an hour to shuffle from the bathroom through to the sitting-room so I could pull myself up on an armchair.”

“Didn’t need to.” [He stood up from a stool after washing his feet in a bowl on the floor and fell backwards. Difficult getting up—pulling himself up by the sink he fell backwards again, but still didn’t use his call alarm]

**Difficulty in activating alarm**

“I couldn’t have reached the alarm . . . like, well, I’ve got the frame but I can’t always reach it.” [Fell trying to get from bed to commode while in hospital]

“I tried to call Care Call but the pendant didn’t work because there’d been a power cut the day before.” [The trolley he was walking with tipped up and he went over too, got himself across floor to climb up on to armchair in about 10 minutes, then waited a couple of hours for his carer to come]

“I was wearing my alarm but I didn’t think it would work out there in the street so I didn’t even try it.” [Lost balance at the gate, fell on pavement outside her flat] “I sat at the kerb trying to get up but I couldn’t. After what seemed like ages a car came by and a man wound down his window to ask if I was okay. He helped me up and back indoors.”

“It always seems a long time when you’re waiting but I don’t suppose it was really.” [Found on floor by carer, confused. She had not set off the alarm, although she thought she had)

## Discussion

### Study summary

In this intensive follow-up of a full survey sample from a population based study of very old people, we found high rates of serious consequences of falling—being unable to get up and thus lying on the floor for a long time, the latter strongly associated with cognitive impairment. Lying on the floor for over an hour was also strongly associated with serious injuries, admissions to hospital, and subsequent moves into long term care. Call alarm systems were widely available but were often not used when they could have been. Virtually no one who did use an alarm lay more than an hour on the floor, yet nearly all (97%) of those who lay on the floor for a long time and had a call alarm system did not activate it. The findings contribute new insights to falls research from a year’s prospective data gathered specifically from people of advanced age (≥90), including men and women living in the community and in supported or institutional settings. The comments of these older old people add a further important element to the findings: their subjective perspective on calling help after falling.

### Limitations

Falls research inevitably faces considerable methodological issues concerning reliable ascertainment, and even in this prospective data collection we had to rely on recall to some extent. None the less, we aimed to maximise validity of the data by deliberately using a combination of methods—participant and proxy reports by calendar, phone calls, and visiting—and achieved remarkably complete data concerning the immediate sequelae of each fall. Despite the high incidence of unwitnessed falls, there was little scope for misreporting the proportion of people unable to get up from the floor unaided, one of the key outcomes in this paper, as these people were by definition still on the floor when help arrived. Length of time on the floor is a less robust measure, for various reasons perhaps overestimated or underestimated by anyone who fell, but possibly also under-reported by proxy informants. As most reports of such falls were provided by carers, the overall effect of any such measurement error is likely to be an underestimate of the extent of this problem.

Some caution is warranted in interpreting the analyses of association between risk factors and these consequences of falling; lack of association might indicate only the limited power of the small sample size. We used a descriptive epidemiological approach examining a range of factors to supplement the dearth of data concerning falls in this age group, as even this small study outnumbers the oldest old subgroups enumerated in previous reports.

### Key findings in context

Four out of five people who fell were unable to get up without help on at least one occasion. This far exceeds reported levels of difficulty regarding people in their 60s, 70s, or even 80s, in which up to a quarter could not manage to get up in clinic test situations^3^ ^22^ ^23^ and between a third to a half could not when they fell in the community.^1^ ^4^ ^18^

Prospective data in our study showed that about a third of those people who couldn’t get up after a fall were on the floor over an hour. The proportion of all falls during follow-up in which this occurred was lower—one in five—but was over a third if the person who fell was alone and could not get up without help. For falls in the community and sheltered housing this proportion was around half. These figures are again far higher than previously reported from younger people. In a UK survey in general practice 16% of those aged over 65 who fell in their own homes lay on the floor for more than an hour.^4^ A US community based study of those aged over 60 who had previously fallen found that one in 10 falls left the faller unable to get up for at least five minutes^24^ and even lower rates have been reported for different time periods.^25^ In institutional settings, we found lying on the floor for a long time was less common.

### Implications

These important findings support an earlier call to include teaching older people how to get up if they fall in preventive initiatives.^26^ Recalled falls and reported limitations with mobility were strongly associated with being unable to get up after falling, suggesting that taking a simple clinical history could help to identify those who might benefit. Such training is rare, despite growing evidence for its effectiveness,^3^ ^27^ ^28^ ^29^ including good evidence from trials for using floor rise training in tailored exercise programmes for fall prevention.^29^

Cognitive impairment was the only characteristic that predicted lying on the floor for a long time, probably indicating that the most cognitively impaired are the least likely to summon help when they cannot get up. This finding, in addition to the known increased risk of falling in cognitively impaired elderly people, supports the argument for more work in this challenging area.^30^ The development of automatic fall detectors that do not rely on the wearer activating them^8^ could help to reduce time on the floor for this vulnerable group.

While difficulty in getting up from the floor is more likely in people in supported living, a finding that would be expected, it is interesting to note that the odds of lying on the floor for a long time were not reduced by the additional level of care. This finding might seem contrary to the results in table 2, which show that most institutional falls led to less than an hour on the floor; the odds ratio in table 4, however, reflects instead the proportion of individuals affected rather than falls. Moreover, this analysis grouped those in care homes with people living in sheltered housing, where lying on the floor for over an hour was as common as in the community. Calling for help can still be problematic in institutional settings: the policy occasionally seen of encouraging residents to wear personal alarms in addition to room call bell systems might have a role to play, and the use of video cameras, as introduced in some US care homes, highlights how technological solutions can raise important ethical questions about the balance between privacy, dignity, safety, and the duty of care. Tele-care solutions, however, should not be viewed as a substitute for hands-on care; one possible strategy to reduce the risk of people lying on the floor for a long time after a fall in care homes might be more frequent checking of residents with dementia.

The fact that nearly all people who couldn’t get up had access to a call alarm system but did not use it to summon help raises important questions for care providers. Only a few studies to date have explored older people’s views on assistive technology devices,^11^ ^12^ some reporting overall positive attitudes,^6^ including specific mention that fall alarms improve confidence,^7^ but there have been studies examining reluctance to seek or accept help.^31^ The findings from recent research exploring attitudes to falls prevention are also pertinent: older people are understandably reluctant to be labelled as at high risk for falling.^32^ ^33^ The emergent themes in this study include older people’s justifiable concern to preserve their independence, both in immediate terms—for example, wanting to get up unassisted—and in terms of the longer view—for example, wanting to avoid admission to hospital. Among those who did not have or did not wear any personal alarm, such devices were not perceived as relevant, usually synonymous with a view that falling was unlikely, a self perception that has been reported in other high risk groups,^34^ ^35^ and similar attitudes have been reported to the use of hip protectors.^36^ There are practical implications for provision of information and training arising from the comments from some of the frailer individuals who tried unsuccessfully to use their alarms, such as ensuring users can actually activate the devices and are aware of their signal range.

Comments from study participants and their relatives revealed pertinent concerns on which further qualitative research might shed more light, particularly to inform the development of interventions to reduce the length of time people lie on the floor after a fall, including the design of call or detector systems. These are complex issues that care providers need to understand better so as to offer and effectively deliver support services and devices that are acceptable to the older people concerned.

**What is already known on this topic**

Being unable to get up after falling can have serious consequences for an old person, especially if they are on the floor a long time

**What this study adds**

Being unable to get up and therefore lying on the floor for a long time are highly prevalent after falls among men and women aged over 90
Severe cognitive impairment was the only intrinsic factor predicting lying on the floor for a long time
Lying on the floor for a long time after a fall was associated with repeated falls, fall induced injuries, and subsequent admissions to hospital and long term care
Alarm systems were widely available but rarely used to call for help to get up

## Notes

We gratefully acknowledge the contributions of previous investigators and past research team members (see www.cc75c.group.cam.ac.uk/pages/studypersonnel), collaborating general practitioners and their practice teams, and, most particularly, the study respondents, their families and friends, and the staff in many care homes. Rosemary Abbott, Stephen Barclay, Tom Dening, Morag Farquhar, Felicia Huppert, Ann-Louise Kinmonth, Fiona Matthews, Elizabeta Mukaetova-Ladinska, Graciela Muniz, Angela O’Sullivan, Eugene Paykel, Kathryn Richardson, and Emily Zhao (current CC75C collaborators) commented on earlier drafts of this paper.

Ethical approval: For each participant written informed consent was obtained either in person or from the proxy informant, as approved by Cambridge research ethics committee.

Provenance and peer review: Not commissioned; externally peer reviewed.

## Notes

**Cite this as: ***BMJ* 2008;337:a2227
